# The Social Skin: A Neuroaesthetic Theoretical Framework for Multimodal Subconscious Signaling in Human Attraction

**DOI:** 10.1093/asjof/ojag186

**Published:** 2026-09-24

**Authors:** Steven H Dayan

## Abstract

First-impression formation in humans occurs within 100 ms and demonstrates remarkable stability across cultures. The present manuscript advances a highly conceptual theoretical framework in which the integument is recognized as a bidirectional sensory-emissive organ instrumental to the first impressions projected and received. At least 5 biologically plausible signaling channels are proposed: (1) thermal infrared radiation; (2) ultraweak photon emission; (3) the cardiac electromagnetic field; (4) volatile chemosignals; and (5) affective touch mediated by C-tactile afferents. A subcortical–cortical integrative network, anchored by the amygdala for rapid salience detection, the insular cortex for interoceptive integration, and the ventromedial prefrontal cortex for value conversion, is proposed as the unifying neural substrate through which these channels converge into the rapid judgments characterized in first-impression research. This integration is proposed as a candidate mechanism that may contribute to the speed and stability of human social recognition, and reframes the dermal and aesthetic interventions performed in cosmetic medicine as impacting a multichannel social signaling network, influencing the psychosocial outcomes increasingly recognized as central to aesthetic medical benefits.

Level of Evidence: 5 (Diagnostic)

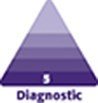

The speed and accuracy with which humans form social judgments have been a recurring subject of psychological investigation. Willis and Todorov demonstrated that exposures as brief as 100 ms are sufficient for stable trait inferences of trustworthiness, competence, and attractiveness.^1^ Ambady and Rosenthal established that judgments derived from sub-30-s silent video clips correlate with longer-term evaluations across professional and interpersonal domains.^2^ Previous work by our group has extended these findings into aesthetic medicine, demonstrating that botulinum toxin treatment of the glabella alters first-impression ratings, that filtered selfies systematically distort self-perception, that video-conferencing modalities modulate self-perceived impressions, and that masking during the COVID-19 pandemic measurably altered face perception.^3-6^

A consistent finding across this literature is that the speed of impression formation exceeds what conscious processing alone could account for. Furthermore, preliminary evidence indicates that visual information may not be necessary for attractiveness judgment. A small pilot study by our group found that visually blind raters detected beauty at levels comparable to blindfolded sighted controls, significantly below that of sighted controls, indicating that nonvisual channels may carry beauty-relevant information. Although caution is advised, the sample was modest and the finding awaits confirmation in larger studies.^7^ These observations raise empirical questions: through what channels, beyond vision, does the social information that drives first impressions and attractiveness travel between humans; and through what neural substrate is that information integrated rapidly enough to produce the first-impression judgments observed?

The idea of the skin as a bidirectional sensory-emissive organ engaged in social signaling has been established across decades of research in human and nonhuman primate biology. The proposition advanced here is that the integument functions not merely as a visible surface nor solely as a barrier organ but as an organ critical to social signaling. Beauty, from an evolutionary biological perspective, has been defined as a subconsciously transmitted form of communication conveying a message of health, wellness, and vitality.^8^ Throughout this manuscript we define beauty as the primitively encoded subconsciously transmitted signal independent of a second individual. In contrast, attractiveness is more evolutionarily advanced and requires both a sender to transmit signals of beauty and a perceiver's evaluative response to that signal. These constructs overlap but are not interchangeable. It should also be emphasized that the author contends that beauty evolved, at least in part, as an honest signal of health, wellness, and underlying genetic fitness. The empirical association between beauty, attractiveness, and physical health nonetheless remains contested. Although a body of evidence suggests facial attractiveness may signal aspects of health, large longitudinal datasets have failed to demonstrate a reliable association between attractiveness and physical health.^9,10^ The present framework treats attractiveness as a probabilistic cue to physiological condition rather than a confirmed index. Five channels are theorized as subconscious mechanisms by which that signaling is transmitted, 4 mediated at a distance and one mediated by contact, each grounded in a wide range of peer-reviewed evidence. The present framework is deliberately scoped to the integument and lesser-known, skin-mediated channels. Although first impressions also encompass rapid judgments of trustworthiness, dominance, competence, warmth, and social approachability, attractiveness is used as the organizing dimension through which the proposed channels are examined. The framework is not intended to generalize to those other social judgments without further study. The subliminal sensing is processed within primitive neural substrates, with particular attention to the amygdala as a subcortical salience detector, the insular cortex as the cortical hub for interoceptive integration, and the ventromedial prefrontal cortex as the site at which integrated signal is converted into the value-based, decision-relevant representations on which humans act. The convergence of these channels at this subcortical–cortical network, rather than any single channel, is proposed as a theoretical mechanism for the rapid social recognition and attractiveness ratings documented in the first-impression literature.

## THE THEORETICAL FRAMEWORK

### Channel I: Thermal Infrared Radiation and Cutaneous Thermoreception

The human body continuously broadcasts heat. Skin behaves as an infrared emitter, and the radiation it produces is not constant: it tracks the underlying physiology of the body, varying with microcirculatory perfusion, sympathetic tone, inflammatory state, fever, and emotional arousal.^11,12^ A person standing at conversational distance is therefore radiating, continuously, a thermal signature of their internal state.

That signal is, in principle, receivable. The molecular substrate within the skin that captures the signal, the transient receptor potential (TRP), transduces both noxious and innocuous thermal stimuli across the cutaneous nerve population.^13,14^ Peripheral thermosensors are activated by changes in their temperature, a mechanism that does not distinguish whether the thermal energy arrived by conduction, convection, or radiation.^15^ The receiving skin is therefore, in principle, equipped to sample the radiated thermal output of another body at close range.

Thermal radiation thus constitutes a candidate channel through which the warmth or coolness of another person could plausibly become a subliminal cue to their vitality and health, and by extension this signal may contribute to the perceived beauty the brain reads from that physiological signal. The sequential steps are individually well established: the body emits infrared radiation continuously, the signal propagates to conversational distance (where it has been reported to be capturable by sensitive instrumentation), and in theory, the TRP channel then receives and transduces the thermal stimuli along with its physiological information. The notion that a person’s emitted infrared is sensed and interpreted by another person at conversational distance has not, to my knowledge, been directly tested but represents a testable prediction of the present framework. What is established is that thermal exchange operates as a social signal across a lifespan, and that, at conversational distance, a direct visual gaze from another can produce measurable shifts in one's facial temperature.^16,17^ An important caveat, however, is that the sensitivity of the thermoreceptors may be insufficient to resolve the minor differences in temperature present at conversational distance.^18^ The proposition that interpersonal thermal signals are perceptually effective at a close distance therefore remains a hypothesis requiring empirical testing.

Level of evidence: Plausible. Thermal emission is well established; reception of another person's radiated heat at conversational distance is biologically plausible but not yet demonstrated and remains a testable prediction.

### Channel II: Ultraweak Photon Emission

Living biological tissue emits photons at extremely low intensities, a phenomenon termed ultraweak photon emission (UPE). Typical emissions fall within the 350 to 1300 nm spectral range, at intensities approximately a thousand times below the threshold of conscious human visual detection.^19,20^ The molecular origin is now well established. Reactive-oxygen species generated during mitochondrial oxidative metabolism produce electronically excited singlet oxygen, which relaxes through photon emission. Cifra and Pospíšil's review provides a synthesis of mechanisms, detection methodologies, and applications.^19^

Human skin UPE has been imaged 2-dimensionally, following a pattern relative to circadian rhythms, and has been shown to correlate with oxidative metabolic status.^21-23^ A systematic review by Ives et al evaluated UPE as a noninvasive health assessment modality and concluded that the signal carries information relevant to physiological state.^24^

The functional significance of UPE in interpersonal signaling remains unproven, and the present framework explicitly designates this channel as the most speculative of the five. The honest position is that UPE is a real and measurable emission whose intensity falls below the threshold of conscious human visual detection. Its inclusion in the framework is justified by its measurable variation with metabolic state that may subconsciously transmit a signal characterizing an individual's health, wellness, and vitality. Critically, however, there is yet to be a known receptor capable of transducing or receiving UPE of this intensity in humans. In the absence of a defined mechanism or comparative precedent, this channel is offered as a conceptual proposition open for direct empirical support.

Level of evidence: Hypothesis-generating (the most speculative of the 5 proposed channels). Emission is established and metabolically linked, but no candidate human receptor capable of transducing photons at this intensity has been identified, and no behavioral demonstration exists.

### Channel III: The Cardiac Electromagnetic Field and Magnetocardiography

Among the body's bioelectric generators, the heart produces the largest field by a substantial margin. The magnetic field generated by cardiac currents is reliably detected outside the body at conversational distance using sufficiently sensitive instrumentation. The clinical modality that measures this magnetic field is magnetocardiography (MCG). Whereas conventional electrocardiography measures the voltage differences that cardiac currents produce on the skin surface, MCG measures the magnetic flux those same electrical currents radiate into the surrounding space. The first human MCG was recorded by Baule and McFee in 1963; the field advanced dramatically with the introduction of superconducting quantum interference device magnetometry published in 1970.^25,26^ Contemporary clinical MCG includes FDA 510(k) clearance for fetal MCG and for noninvasive diagnosis of myocardial ischemia.^27,28^ Accumulated diagnostic data on coronary artery disease and cardiac arrhythmias have been summarized in reviews.^29^

The relevance of MCG to the present framework is established by its clinical-grade measurability and its propagation beyond the body surface. Whether the cardiac field of one individual is biologically received and integrated by a second individual at conversational distance remains a question. McCraty et al have demonstrated that the cardiac electrical signal of one person can be registered in the electroencephalographic recordings of a second person when the 2 are in physical proximity.^30^ A recent independent review proposed a role for the heart–brain axis in interpersonal emotional dynamics and framed the biofield as both a measurable biomarker and an emerging therapeutic target.^31^ Although the notion that we can transmit and receive heart signals from others and have these influence perception and behavior is provocative, it still requires interpretation with prudent skepticism. The cardiac field's existence and clinical measurability are well established, and its biological reception by a second person is recordable and has been replicated. However, there is yet to be a specialized magnetoreceptive organ identified in humans, and the mechanism by which such a field might be transduced remains undefined. However, as an additional signaling channel for interpersonal communication, it is consistent with the subconscious integration that the framework proposes. It is therefore offered as a biologically plausible signaling pathway for messaging emotions, health, wellness, and vitality and is worthy of investigation.

Level of evidence: Hypothesis-generating. Emission and extracorporeal propagation are clinically established through FDA-cleared MCG, and interindividual registration has been recorded; however, no human magnetoreceptor or transduction mechanism has been identified.

### Channel IV: Volatile Chemosignals

The classical mammalian pheromone pathway, mediated by the vomeronasal organ is controversial and arguably regarded as vestigial in humans. However, a more general and richer chemosignaling channel operates through volatile organic compounds emitted from skin, breath, and sweat, decoded through the olfactory epithelium and the trigeminal system.

The de Groot laboratory at Utrecht has produced the most rigorous experimental demonstrations of human emotional chemosignaling. In a 2012 *Psychological Science* publication, de Groot et al demonstrated that women exposed in a double-blind paradigm to sweat collected from men in fear-induced and disgust-induced emotional states produced facial expressions and behaviors congruent with the donor emotion, despite being unaware that any chemical signal was being delivered.^32^ Subsequent work has extended these findings to include happiness.^33^

This channel has similarities to the popularized pheromone framework but remains distinct in several respects. The signal is multimolecular rather than mediated by a small number of dedicated ligands. Receiver decoding occurs through the olfactory and trigeminal pathways rather than a specialized accessory system, and the resulting behavioral effects are best characterized as emotional contagion rather than fixed action patterns. For the purposes of the present framework, the chemosignaling channel represents the best-established nonvisual route by which humans transmit and receive subconscious social information, as well as health-related signaling at conversational distance.

Level of evidence: Supported. Both emission and behavioral/neural reception of human chemosignals are demonstrated in controlled, double-blind experimental studies.

### Channel V: C-Tactile Afferents and Affective Touch

During physical contact a dedicated sensory channel is engaged that is distinct from the discriminative-touch system. Human skin is innervated by slow-conducting, unmyelinated C-tactile (CT) afferents that respond optimally to gentle stroking with velocities in the range of ∼1 to 10 cm/s.^34^ These fibers do not project predominantly to primary somatosensory cortex; instead, their central targets include the posterior insula, orbitofrontal cortex, and other regions of the brain's affective and reward circuitry.^35,36^

McGlone et al's 2014 *Neuron* review provides a synthesis of decades of neuroimaging and behavioral work, concluding that the CT nerve fiber system is modulated and mediated by oxytocin among other neurotransmitters and represents a neurobiological substrate for the affective and rewarding properties of social gentle touch.^34^ This finding reframes the skin from a passive receptive surface to an active sensor whose architecture includes a channel for socially salient contact tuned to affectionate, empathic touch. Much of the CT-affective-touch literature has characterized this system within bonds between mates, kin, and in-group members. The subconscious social influence of touch, however, is not confined to such bonds. Brief, incidental contact between strangers can exert measurable and unconscious effects on behavior. In the now-classic demonstration of the “Midas touch,” a fleeting touch from a server significantly increased restaurant tipping, with patrons unaware that they had been touched or that it had influenced their behavior.^37^ That a momentary tactile cue from a nonintimate can shift social valuation beneath the level of awareness is the effect this framework anticipates. Therefore, it is proposed that affective touch contributes not only to the deepening and maintenance of established bonds but also to the subconscious social evaluation through which affiliation and, plausibly, attraction may first take hold.

Level of evidence: Supported. Affective touch mediated by CT afferents is characterized by direct microneurographic, neuroimaging, and behavioral evidence.

### The Subcortical–Cortical Integrative Network

The framework presented above raises a structural question. If first-impression formation involves the integration of 5 disparate signal streams arriving across radiative, photonic, electromagnetic, chemical, and mechanical modalities, where in the nervous system does that integration occur, and through what mechanism does it produce judgments rapidly enough to satisfy the temporal constraints established in the behavioral literature at a subconscious level? Can it also be theorized that these pathways, when integrated in the subcortical brain, are sensed as beauty (health and vitality) and then interpreted in the cortical substrates as attractiveness? The answer advanced here is that the 5 channels converge on a subcortical–cortical network in which the amygdala serves as the rapid first-pass filter, with the insular cortex and ventromedial prefrontal cortex operating as downstream nodes for interoceptive integration and value conversion ultimately contributing to a perceived degree of attractiveness.

### The Amygdala and Rapid Salience Detection

Within the limbic system, the amygdala is the most thoroughly characterized substrate for the rapid evaluation of biologically salient stimuli. LeDoux established that the amygdala receives sensory input through 2 pathways: a slower, evolutionarily advanced cortical route supporting detailed perceptual analysis and a faster, subcortical “low road” directly from the midbrain without previous cortical processing.^38^ The low road triggers autonomic and behavioral responses before higher-order conscious recognition is complete.

The amygdala has often been called a “fear center,” but the modern view is broader. Phelps and LeDoux describe it as a detector that flags any stimulus the organism must prioritize.^39^ Amygdala activation is elicited not only by threatening stimuli but also by stimuli of high reward value, including attractiveness and untrustworthiness.^40^ Winston et al showed the amygdala responds to facial attractiveness.^41^ Cloutier et al confirmed amygdala engagement by attractive faces along with the orbitofrontal and other reward-related regions.^42^ Freeman et al showed the amygdala responds to social information such as trustworthiness from faces presented too briefly to be consciously seen.^43^ Clinically, these findings are noteworthy to aesthetic medicine because they support the notion that facial appearances send signals that are received by an observer below their conscious awareness.

The implication for the present framework is that the 2 oldest categories of biologically significant social stimulus, threat and reward, both phylogenetically preserved and critical to species survival, are processed immediately in the subcortical brain. The amygdala receives heavy afferent input from the olfactory system, maintains dense reciprocal connections with the insular cortex, and projects forward to the ventromedial prefrontal cortex through which subsequent value computation occurs.^38,39^ The amygdala is, in effect, the network's rapid first-pass filter, a structure with an evolutionary purpose to flag, within milliseconds, the social signals that should be prioritized for health, mating, and survival. This bidirectional relationship between skin, attractiveness, and mood supports previous clinical investigations into the importance and value of the psycho–social–dermal axis.^44^

From the amygdala, the integrated signals project to 2 downstream nodes, the insular cortex, which transforms autonomic and visceral correlates into the felt sense of bodily and social state, and the ventromedial prefrontal cortex, which converts that interoceptive representation into a value-based signal.^45,46^ The amygdala–insula–vmPFC axis thus provides a plausible neural pipeline from rapid subcortical detection to conscious social evaluation.

### Multimodal Integration and Implications for First-Impression and Attractiveness Research

No single channel reviewed above suffices, alone, to account for the speed, stability, and modality of human first impressions. Taken collectively, however, and integrated through the skin–subcortical-brain network, the 5 channels constitute a redundant, multimodal stream of weak signals available to the perceiver at conversational distance. The convergence of these streams on a subcortical–cortical (subconscious–conscious) system, whose components operate on the level of the autonomic nervous system rather than that of cortical deliberation, provides a plausible but still highly conceptual neural substrate for the subconscious recognition processes that first-impression and beauty-perception research has documented behaviorally. These channels, and the markedly different levels of evidence supporting each, are summarized in Table 1.

**Table 1.** Proposed Signaling Channels Stratified by Biological Emission, Evidence of Emission, Evidence of Reception, and Overall Level of Support

Channel	Biological emission	Evidence of emission	Evidence of reception	Overall level of support
Thermal infrared radiation	Skin continuously emits infrared radiation that tracks perfusion, autonomic tone, and arousal	Strong—directly measured by infrared thermography	Limited—cutaneous TRP thermoreceptors can transduce radiant heat, but perceptual sensitivity at conversational distance is unproven	Plausible
Ultraweak photon emission	Metabolic, reactive-oxygen-species–driven photon emission from skin	Strong—imaged, circadian, linked to oxidative metabolism	None established—no candidate human receptor at these intensities	Hypothesis-generating (most speculative)
Cardiac electromagnetic field	Heart generates a magnetic field that propagates beyond the body surface	Strong—FDA-cleared magnetocardiography	Weak—interindividual registration recorded, but no human magnetoreceptor identified	Hypothesis-generating
Volatile chemosignals	Volatile organic compounds from skin, breath, and sweat carrying emotional and physiological state	Strong—collected and chemically characterized	Strong—double-blind transfer of donor emotional state, behavioral, and neural	Supported
Affective touch (C-tactile afferents)	Gentle skin-to-skin contact activating C-tactile afferents	Strong—microneurographic recordings	Strong—C-tactile projection to insula and orbitofrontal cortex; behavioral effects (eg, the “Midas touch”)	Supported

Levels of support are defined as supported (direct experimental evidence for both emission and reception), plausible (emission established, reception biologically plausible but not yet demonstrated), and hypothesis-generating (emission established, reception not demonstrated, and no receptor mechanism identified). TRP, transient receptor potential; FDA, US Food and Drug Administration.

The framework generates testable predictions for future investigation. First, experimental disruption of any single channel should degrade but not eliminate first-impression accuracy, with the magnitude of degradation reflecting the contribution of that channel to overall integration. Second, interventions that modulate the emission profile of a channel, such as antiperspirants modifying volatile chemosignals, vasoactive interventions modifying thermal radiation, or autonomic regulations modifying cardiac signal coherence, may produce measurable effects on third-party social judgments. Third, individuals with clinically characterized interoceptive deficits, including those documented in alexithymia (“emotional blindness”) and in certain anxiety disorders, should demonstrate altered first-impression performance specifically in tasks requiring integration across nonvisual modalities. Finally, dampening of reward-center activity, as may occur with the new class of glucagon-like peptide-1 receptor agonists (GLP-1s), may plausibly influence the valuation of beauty perception, an empirical question that warrants direct investigation.

A further corollary concerns visually blind individuals, who should, if the framework is directionally correct, demonstrate greater integration efficiency across nonvisual channels than blindfolded sighted controls. Provocative, highly preliminary evidence from our pilot observational study showed that blind raters detected beauty at levels comparable to blindfolded sighted controls, consistent with the basic claim that nonvisual channels carry beauty-relevant information, but the sample was small and larger, methodologically rigorous studies are required before strong inferences can be drawn.^7^

### Implications for Aesthetic Medicine

If the framework is directionally correct, several reorientations of aesthetic and dermatologic practice follow. The first is conceptual. The skin upon which we operate, inject, peel, laser, and treat topically is not solely a visible surface; it is a multichannel social signaling organ that we may alter with interventions. Aesthetic treatments, accordingly, are not solely a modification of appearance; they are a modification of the patient's broadcast and receiver across multiple subconscious channels.

The second is empirical. The well-documented psychosocial benefits of aesthetic interventions include improvements in confidence, professional outcomes, perceived warmth and approachability, and the bidirectional relationship between skin condition and mood.^44,47^ These benefits become substantially more significant when the treated skin is understood as participating in subconscious interpersonal signaling. The framework presented offers a theoretical neuroaesthetic mechanism that may help account for outcomes that have historically been described as psychological rather than cosmetic or biological.

The third is therapeutic. If skin both broadcasts and receives, then aesthetic outcomes should be evaluated not only by appearance endpoints but also by scales evaluating the first-impression broadcast and received as well as the psychosocial benefits resulting from the interventions employed. Validated instruments exist for capturing some of these constructs; the FACE-Q Aesthetics Natural Module moves the field in this direction.^48^ Future evaluations will require aesthetic treatments and products to go beyond patient-reported and observer-reported outcomes to demonstrate the social currency an individual gains from cosmetic interventions.

### Limitations

The framework presented here is theoretical and intended to provide a neuroaesthetic scaffolding for understanding the psychosocial impact aesthetic medical interventions have on patients and on those with whom they interact. The 5 channels are established individually in their respective literatures, but the proposition that they are jointly integrated through the subcortical primitive brain and reward-center network to constitute the operative mechanism of first-impression formation is, at present, a highly theoretical hypothesis intended to stimulate thought and generate testable predictions rather than a settled finding. The framework proposes a model that further explains the multilevel impact cosmetic interventions may have on the impressions an individual projects. The concepts presented should not be read as endorsing claims of paranormal communication or other phenomena outside conventional biophysics. The channels described are all physically plausible, operate within established sensory ranges, and converge on a neural network that is well characterized in the neuroscience of detection, interoception, value, emotion, and social cognition. Finally, the framework should not be interpreted as implying aesthetic interventions directly improve health through appearance. The association between attractiveness and health remains contested. The framework concerns the signaling and social-perceptual consequences of appearance and should not be misconstrued as ascribing biological superiority or greater health to any individual or group.

## CONCLUSIONS

The body's largest organ, the skin, serves as a bidirectional sensory-emissive organ that may participate in subconscious social signaling of health, wellness, and vitality through at least 5 proposed channels: thermal radiation, UPE, the cardiac electromagnetic field, volatile chemosignals, and affective touch. Beauty, attractiveness, and outcomes from cosmetic interventions may be projected, received, and processed through channels broader than vision alone, carrying psychosocial value that assessments by appearance may not capture. Evaluating outcomes in aesthetics by 2-dimensional photography alone neglects the breadth or influence a cosmetic intervention has on a patient and the social currency gained from the product or procedure employed. By recognizing the convergence of the 5 subconscious channels of communication at a subcortical–cortical network, the framework offers a conceptual model that may help to account for the rapid social judgments documented in first-impression research. Further empirical work is required to test the framework's specific predictions and to translate it into clinical practice.
